# Evidence-based guidelines and protocols for the management of adult patients with a tracheostomy: a systematic review

**DOI:** 10.1186/cc12104

**Published:** 2013-03-19

**Authors:** J Siddiqui, PB Sherren, MA Birchall

**Affiliations:** 1 North West London Hospitals NHS Trust, London, UK; 2 Barts and The London NHS Trust, London, UK; 3 The Royal National Throat Nose and Ear Hospital, London, UK

## Introduction

Protocol-based care of the tracheostomised patient is important, as adverse events confer a high rate of mortality. Little is known regarding the existence of formal evidence-based guidelines on tracheostomy care. The aim of this study was to perform a systematic review for evidence-based guidelines on adult tracheostomy care.

## Methods

A systematic search of PubMed, MEDLINE, guideline clearinghouses, centres of evidence-based practice, and professional societies' guidelines relating to care of adult patients with a tracheostomy was performed by two reviewers. In addition, a Google search of publicly available tracheostomy care guidelines was performed. Search terms: (tracheostom* OR tracheotom*) AND (protocol* OR guideline* OR standard* OR management OR consensus OR algorithm*). Filters: English language, human, from 1 January 1990 to date, adult patients. Guideline appraisal criteria: the quality of guidelines retrieved was assessed using the Appraisal of Guidelines Research and Evaluation II (AGREE II) instrument [1].

## Results

The search results are summarised in Table 1. A total of 80 guidelines were identified. Five were found to satisfy the AGREE II criteria and only three related to the entire spectrum of tracheostomy management. The majority was informal and was not published or evidence based.

**Table 1:** Summary of results returned following systematic review

Database	Total results	Results with limits	Full texts retrieved	Guidelines found	AGREE instrument compliant
PubMed	4,685	1,596	33		
MEDLINE	3,859	1,337	17	26	2
CINAHL	711	172	6		
Medical societies/guideline websites/clearinghouses	N/A	N/A	N/A	54	3
Google	17,600,000	497	N/A		

## Conclusion

Five evidence-based guidelines on adult tracheostomy management were identified. This may represent a paucity of evidence on the subject, suggesting that further clinical trials on the topic are needed to contribute to the evidence base. This also highlights the need for international consensus on the topic, to reduce duplication of efforts, standardise practice, and improve outcomes.
